# Positive Affective Recovery in Daily Life as a Momentary Mechanism Across Subclinical and Clinical Stages of Mental Disorder: Experience Sampling Study

**DOI:** 10.2196/37394

**Published:** 2022-11-23

**Authors:** Leonie Ader, Anita Schick, Claudia Simons, Philippe Delespaul, Inez Myin-Germeys, Thomas Vaessen, Ulrich Reininghaus

**Affiliations:** 1 Department of Public Mental Health, Central Institute of Mental Health, Medical Faculty Mannheim Heidelberg University Mannheim Germany; 2 Department of Psychiatry and Neuropsychology, School for Mental Health and Neuroscience, Faculty of Health, Medicine and Life Sciences Maastricht University Maastricht Netherlands; 3 GGzE Institute for Mental Health Care Eindhoven Netherlands; 4 Department of Adult Psychiatry, Mondriaan Mental Health Trust Heerlen Netherlands; 5 Center for Contextual Psychiatry, Department of Neuroscience KU Leuven Leuven Belgium; 6 Faculty of Behavioural, Management and Social Sciences, Psychology, Health & Technology University of Twente Enschede Netherlands; 7 ESRC Centre for Society and Mental Health King's College London London United Kingdom; 8 Centre for Epidemiology and Public Health, Health Service and Population Research Department, Institute of Psychiatry, Psychology & Neuroscience King’s College London London United Kingdom

**Keywords:** experience sampling methodology, ecological momentary assessment, trajectory, transdiagnostic, resilience, stress reactivity, psychosis, depression

## Abstract

**Background:**

Identifying momentary risk and protective mechanisms may enhance our understanding and treatment of mental disorders. Affective stress reactivity is one mechanism that has been reported to be altered in individuals with early and later stages of mental disorder. Additionally, initial evidence suggests individuals with early and enduring psychosis may have an extended recovery period of negative affect in response to daily stressors (ie, a longer duration until affect reaches baseline levels after stress), but evidence on positive affective recovery as a putative protective mechanism remains limited.

**Objective:**

This study aimed to investigate trajectories of positive affect in response to stress across the continuum of mental disorder in a transdiagnostic sample.

**Methods:**

Using the Experience Sampling Method, minor activity-, event-, and overall stress and positive affect were assessed 10 times a day, with time points approximately 90 minutes apart on six consecutive days in a pooled data set including 367 individuals with a mental disorder, 217 individuals at risk for a severe mental disorder, and 227 controls. Multilevel analysis and linear contrasts were used to investigate trajectories of positive affect within and between groups.

**Results:**

Baseline positive affect differed across groups, and we observed stress reactivity in positive affect within each group. We found evidence for positive affective recovery after reporting activity- or overall stress within each group. While controls recovered to baseline positive affect about 90 minutes after stress, patients and at-risk individuals required about 180 minutes to recover. However, between-group differences in the affective recovery period fell short of significance (all *P*>.05).

**Conclusions:**

The results provide first evidence that positive affective recovery may be relevant within transdiagnostic subclinical and clinical stages of mental disorder, suggesting that it may be a potential target for mobile health interventions fostering resilience in daily life.

## Introduction

When developing a mental disorder, an individual is commonly assumed to experience a state in which psychological distress and symptoms gradually increase without fully meeting diagnostic criteria [1,2]. Corresponding to staging models in general medicine, the concept of clinical staging in psychiatry broadens the dichotomous definition of mental health versus ill-health by placing an individual on a continuum that defines thresholds for different stages of mental disorders [3-5]. Especially the identification of early stages of mental disorder marked by psychometric and familial risk criteria has received increasing attention as a potential target group for early intervention and prevention programs [4,6]. Psychometric risk states can be characterized by nonspecific distress and attenuated symptoms that are not disorder specific, thereby implying a transdiagnostic perspective on early stages of mental disorders [4,6,7]. In addition, there is evidence for an increased familial liability to severe mental disorders, such as psychosis [8] and major depression [9,10], suggesting that even relatives without a formal diagnosis of a disorder can be placed closer toward clinical thresholds on the continuum of mental health.

There is consistent evidence on high comorbidity in at-risk individuals, which has been taken to suggest a pluripotent risk state or early shared mechanisms, from which individuals may transition to different, more specific exit syndromes of severe mental disorder, for example, psychotic or affective disorders [1,3,11]. One common underlying mechanism that has been proposed is behavioral sensitization. Specifically, it has been posited that, in individuals exposed to severe and repeated adversity across the life course, the stress response is gradually amplified such that they eventually show a strong response to even minor stressors in daily life [12], which may, in turn, be associated with a greater risk of transitioning to mental disorder. The most commonly used behavioral marker of stress sensitization is elevated stress reactivity, characterized by strong emotional reactions to minor stressors in daily life (eg, [12-15]), measured with experience sampling methodology (ESM), an intensive longitudinal diary technique [16]. Indeed, stress reactivity has been found to be elevated in individuals with an increased risk for [17,18] and a diagnosis of severe mental disorder [13,15,17]. Furthermore, there is evidence pointing toward stress reactivity measured in experience sampling studies being more pronounced in at-risk individuals than in patients [13,18-20].

Focusing on underlying mechanisms, experience sampling studies have emphasized the importance of investigating risk and resilience mechanisms when studying transdiagnostic and subclinical samples in daily life [21,22]. Resilience has been defined as the ability to recover from the effects of significant adversity [23,24]. Translating this definition to the realm of momentary mechanisms measured with experience sampling, it is tempting to speculate whether momentary resilience may be reflected in the ability to recover, in the moment, from minor stressors and adverse experiences in daily life.

So far, research into momentary mechanisms has focused on negative affect. There is initial evidence that individuals with early mental health problems may experience extended momentary negative affective recovery from minor stressors in daily life, that is, they take longer to overcome minor adversities in daily life [20]. Indeed, positive affect has been proposed as an important building block of resilience [25,26] that can be relevant when recovering from negative experiences [24,27]. Importantly, patients (see [28]), but also individuals at-risk for mental disorder (eg, [29,30]), have been shown to be less sensitive to positive stimuli and may have a reduced ability to experience positive emotions overall (ie, anhedonia), suggesting that they may potentially show different trajectories of positive affect after experiencing stressors.

Against this background, this study aimed to investigate trajectories of momentary positive affect following exposure to minor stressors in daily life across transdiagnostic stages of mental disorder in a pooled sample of patients with a mental disorder (ie, psychotic disorder, depressive disorder with residual symptoms), individuals with an increased psychometric or familial risk for developing a severe mental disorder, and controls. To examine, in detail, the entire positive affective recovery process from minor stressors through to recovery to baseline levels, we aimed to investigate (1) levels of positive affect prior to reporting a minor daily stressor; (2) initial positive affective reactivity following the stressor—operationalized as the decrease in positive affect associated with minor (i) event-related, (ii) activity-related, and (iii) composite stress (as previously operationalized in experience sampling studies [21,31,32]); and (3) positive affective recovery from stress—operationalized as the average decrease of positive affect from baseline across the period between the occurrence of minor stress and return to baseline. Echoing previous findings that individuals with early stages of mental disorder experience the most pronounced reactions related to stress, marked by reactivity [13,18-20] and negative affective recovery [20], compared with patients with an enduring mental disorder, we aimed to investigate group differences between at-risk individuals and patients. Specifically, we sought to test the following hypotheses (see Multimedia Appendix 1):

H1: Within each group (patients with a mental disorder, at-risk individuals, controls), exposure to (i) event-related, (ii) activity-related, or (iii) composite stress is associated with (a) an initial decrease in positive affect (ie, stress reactivity) and (b) subsequent to initial stress reactivity, lower levels of positive affect before recovering to baseline level (ie, affective recovery).

H2: Baseline levels of positive affect, that is, prior to reporting (i) event-related, (ii) activity-related, or (iii) composite stress, are lower in (a) patients with a mental disorder than in controls, (b) at-risk individuals than in controls, and (c) at-risk individuals than in patients with a mental disorder.

H3: Positive affective reactivity from minor stress is greater in (a) patients with a mental disorder than in controls, (b) at-risk individuals than in controls, and (c) at-risk individuals than in patients with a mental disorder.

H4: Positive affective recovery from minor stress, that is, the average decrease of positive affect from baseline before returning to baseline levels of positive affect following (i) event-related, (ii) activity-related, or (iii) composite stress, is greater in (a) patients with a mental disorder than in controls, (b) at-risk individuals than in controls, and (c) at-risk individuals than in patients with a mental disorder.

## Methods

### Samples

The pooled sample comprised participants from 8 previously conducted studies that used a similar protocol and are part of the ESM merge file. These studies included individuals with a mental disorder, that is, psychotic disorder [17,33-38] or depressive disorder with residual symptoms [39]; at-risk individuals, that is, with familial [17,34,36,40] or psychometric risk for psychosis [19,38]; and controls without a personal or family history of mental disorder [17,19,34,36,38,40]. The samples and procedures to obtain diagnoses and risk status of the participants have been described elsewhere (see Multimedia Appendix 2).

### Ethical Approval

All 8 studies received approval by their respective medical ethics committees in the Netherlands and Belgium as stated in the original references and all procedures were performed in accordance with the ethical standards of the responsible medical ethics committee. This study was registered on OSF (Open Science Framework) before data access [41].

### Data Collection

#### Experience Sampling Method

Data were collected using the ESM, a structured diary technique [16,42]. Participants received a digital wristwatch that sent 10 signals per day at pseudo-random time points in blocks of 90 minutes between 7.30 AM and 10.30 PM for 6 consecutive days. The signal prompted participants to complete questionnaires on their current mood, symptoms, and context that they had previously received in a booklet. To ensure compliance with the experience sampling procedure, only prompts answered within 15 minutes after the programmed signal and participants who answered a minimum of 20 prompts were included in the analysis.

#### ESM Measures

For the current analysis, experience sampling constructs available in all included studies were selected to measure positive affect, momentary event-related stress, and momentary activity-related stress. Positive affect was measured with 3 items beginning with “I feel” followed by the adjectives “cheerful,” “relaxed,” and “satisfied” (1=not at all; 7=very much). Based on previous experience sampling studies [15,17,18], momentary stress was operationalized by 2 types of minor stressors. Event-related stress was measured by asking about the most important event for the participant that happened since the last prompt. Participants then indicated how pleasant this event was on a bipolar scale (–3=very unpleasant; 3=very pleasant, which was recoded to 1=very pleasant to 7=very unpleasant, to match the other scales). To measure activity-related stress, participants were asked what they were doing at the moment followed by 4 questions on their current activity: “This costs energy,” “I’m skilled at this” (reverse coded), “This is a challenge,” and “I prefer doing something else” (1=not at all; 7=very much).

Mean scores of the 3 positive affect items were centered around the person and day means and z standardized. In addition to momentary event– and activity-related stress, after justifying its use by principal component analysis (see Multimedia Appendix 3), a composite stress measure indicating the presence of one or both types of stress combined (0=no stress; 1=one or both types of stress) was created (see [21,31,32]). Individuals who never reported stress and days on which no stress was reported were excluded from the analysis.

### Statistical Analysis

Stata version 16.0 (StataCorp LLC) was used for statistical analysis [43]. Experience sampling data have a 3-level structure with individual assessments (level 1) nested within days (level 2), which are, in turn, nested within individuals (level 3). Group differences on level 3 variables (ie, age and gender) were examined using 1-way ANOVAs and chi-square tests as appropriate, whereas group differences on levels 1 and 2 were examined using Stata’s “mixed” command for multilevel models.

To test the hypotheses, the procedure described by Vaessen et al [20] was followed. Trajectories of positive affect in response to the first stressor of a day were examined to rule out the potential cumulative impact of consecutive stressors throughout a day on positive affect. A new predictor variable “time_since” was created for each stress measure to mark the time points when positive affect was measured in relation to the first stressor of the day. The time point when the stressor occurred (ie, stress reactivity) was set to t_0_, the time point prior to this (ie, t_–1_) served as the baseline, and all time points following the stressor were set to t_1–n_. First, to test H1, in a separate model for each group using time_since to predict positive affect, we compared all time points t_0–n_ with baseline. Second, to test H2, group was added as a predictor in the model and group comparisons of positive affect were calculated at baseline and t_0_. Third, to test H3, an interaction between time_since and group was specified in the model to compare affective reactivity at t_0_ between groups. Last, affective recovery was compared between groups (H4) using the average decrease of positive affect from baseline across the recovery period. Specifically, a recovery period of 2 prompts was specified as the average deviation of positive affect at these time points from baseline positive affect.

For each momentary stressor (event-related, activity-related, and composite stress), separate models were fitted. For each model, observations were excluded (i) for participants who never reported the specific type of stress, (ii) for days on which the specific type of stress was not reported, and (iii) for days on which the specific type of stress was reported on the first prompt of the day so that no baseline measure was available.

All models were adjusted for age (centered using the grand mean) and gender (for unadjusted models, see Multimedia Appendix 4). As a sensitivity analysis, the analysis was repeated controlling for subsequent stressors. To this end, dichotomous control variables were created for event- and activity-related, or composite stress indicating the presence (=1) or absence (=0) of the respective stressor at all time points t_n>0_. We used Simes correction [44] to account for multiple tests of significance regarding our 3 stress measures, as all models testing our specific hypotheses were repeated for each stress measure. Therefore, according to the Simes procedure, the most significant *P* value within each model was compared with α=.05/3=.02 and the second most significant *P* value was compared with α=.05/2=.03. Results that remain significant after Simes correction are marked with footnotes in tables. A significance level of *P*<.05 was set for all remaining *P* values.

## Results

### Sample Characteristics

The sample comprised 921 participants. This includes 422 individuals with a mental disorder (ie, 293 with psychotic disorder and 129 with remitted depressive disorder with residual symptomatology), 246 at-risk individuals (ie, 178 with familial and 68 with psychometric risk), and 253 controls. Participants completed a total of 42,778 prompts. Average compliance was 75% (45/60 prompts) for patients, 78% (47/60 prompts) for at-risk individuals, and 82% (49/60 prompts) for controls (*F*_2,918_=13.02, *P*<.001). Across groups, 2304 prompts were not completed within 15 minutes after the signal or all positive affect and stress items were missing (*χ*^2^_2_=21.2, *P*<.001). In addition, 34 participants completed less than 20 prompts over 6 days (*χ*^2^_2_=2.8, *P*=.24) and 75 participants never reported any type of stress (*χ*^2^_2_=0.01, *P*=.95) and were therefore excluded from the analysis.

Hence, the analytic sample consisted of 811 participants (patients/at-risk/controls: n=367/217/227) with a total of 39,903 valid prompts (patients/at-risk/controls: n=16,122/9997/10,784). Sample characteristics of the analytic sample are depicted in Table 1.

**Table 1:** Basic sample characteristics.

Characteristic		Patients (n=367)	At-risk (n=217)	Controls (n=227)	Test statistic	*P* value	Significant contrasts
**Gender, n**					*χ*^2^_2_=7.7	.02	
	Male	187	90	93			
	Female	180	127	134			
Age, mean (SD)		38.07 (11.42)	36.41 (13.12)	35.50 (12.56)	*F*_2,806_=3.4	.04	Patients versus controls
Observations per person, mean (SD)		43.93 (10.07)	46.07 (9.21)	47.51 (9.10)	*F*_2,808_=10.3	<.001	Patients versus controls
Stressful days per person, mean (SD)		5.92 (0.58)	5.96 (0.41)	5.99 (0.48)	*F*_2,808_=1.4	.25	
Time of first stressor^a^, mean		2:59 PM	3:07 PM	2:55 PM	*F*_2,769_=0.3	.72	
Unpleasantness of first stressor^a^, mean (SD)		2.00 (0.62)	1.88 (0.63)	1.91 (0.63)	*F*_2,770_=3.1	.04	Patients versus at-risk
Positive affect, mean (SD)		4.27 (0.93)	4.89 (0.94)	5.16 (0.71)	*F*_2,808_=79.4	<.001	At-risk versus controls; patients versus controls; patients versus at-risk

^a^Excluding stressors that were reported at the first prompt of the day.

### Recovery Period Within Groups (H1)

#### Patients

Patients showed a decrease in positive affect in response to all types of stress (event-related stress: *b*=–0.35, 95% CI –0.43 to –0.28, *P*<.001; activity-related stress: *b*=–0.49, 95% CI –0.60 to –0.38, *P*<.001; composite stress: *b*=–0.38, 95% CI –0.45 to –0.31, *P*<.001). Following event-related stress, recovery occurred at t_1_, that is, patients had immediately returned to baseline levels of positive affect (*b*=–0.06, 95% CI –0.13 to 0.02, *P*=.16). Following activity-related (*b*=–0.14, 95% CI –0.26 to –0.02, *P*=.02) and composite stress (*b*=–0.11, 95% CI –0.19 to –0.04, *P*<.01), patients still showed a significant decrease at t_1_. At t_2_, patients also had returned to baseline levels of positive affect following activity-related stress (*b*=0.01, 95% CI –0.12 to 0.13, *P*=.90) and composite stress (*b*=–0.01, 95% CI –0.09 to 0.06, *P*=.71).

#### At-Risk Individuals

At-risk individuals showed a decrease in positive affect in response to all types of stress (event-related stress: *b*=–0.34, 95% CI –0.43 to –0.26, *P*<.001; activity-related stress: *b*=–0.54, 95% CI –0.68 to –0.40, *P*<.001; composite stress: *b*=–0.38, 95% CI –0.46 to –0.30, *P*<.001). Following event-related stress, recovery occurred at t_1_, that is, at-risk individuals had immediately returned to baseline levels of positive affect (*b*=–0.75, 95% CI –0.16 to 0.02, *P*=.10). Following activity-related (*b*=–0.17, 95% CI –0.33 to –0.02, *P*=.03) and composite stress (*b*=–0.11, 95% CI –0.20 to –0.03, *P*=.01), at-risk individuals still showed a significant decrease at t_1_. At t_2_, at-risk individuals also had returned to baseline levels of positive affect following activity-related stress (*b*=–0.09, 95% CI –0.25 to 0.07, *P*=.27) and composite stress (*b*=–0.05, 95% CI –0.14 to 0.03, *P*=.23).

#### Controls

As with the other groups, controls showed a decrease in positive affect in response to all types of stress (event-related stress: *b*=–0.27, 95% CI –0.35 to –0.19, *P*<.001; activity-related stress: *b*=–0.60, 95% CI –0.74 to –0.46, *P*<.001; composite stress: *b*=–0.32, 95% CI –0.40 to –0.25, *P*<.001). Similar to patients and at-risk individuals, controls returned to baseline levels of positive affect immediately at t_1_ following event-related stress (*b*=–0.04, 95% CI –0.13 to 0.04, *P*=.32). Controls had also recovered immediately at t_1_ following activity-related (*b*=–0.15, 95% CI –0.30 to 0.001, *P*=.05) and composite stress (*b*=–0.07, 95% CI –0.15 to 0.01, *P*=.10; Table 2).

**Table 2:** Within-group analysis of all stress measures comparing positive affect at baseline (t_–1_) with time points t_0_ (stress reactivity), t_1_, and t_2_ (all groups recovered) adjusted for age and gender^a^.

Stress type			Patients					At-risk					Controls				
			*b* (CI)		*P* value		*b* (CI)			*P* value		*b* (CI)			*P* value		
**Event-related stress^b^**																	
	t_0_	–0.35 (–0.43 to –0.28)		<.001^c^		–0.34 (–0.43 to –0.26)			<.001^c^		–0.27 (–0.35 to –0.19)			<.001^c^			
	t_1_	–0.06 (–0.13 to 0.02)		.16		–0.08 (–0.16 to 0.02)			.10		–0.04 (–0.13 to 0.04)			.32			
	Age	–0.002 (–0.01 to 0.01)		.52		0.01 (0.002 to 0.02)			.01^c^		0.01 (0.004 to 0.02)			.001^c^			
	Gender	–0.13 (–0.28 to 0.03)		.11		0.06 (–0.14 to 0.27)			.56		0.001 (–0.16 to 0.16)			.99			
**Activity-related stress^d^**																	
	t_0_	–0.49 (–0.60 to –0.38)		<.001^c^		–0.54 (–0.68 to –0.40)			<.001^c^		–0.60 (–0.74 to –0.46)			<.001^c^			
	t_1_	–0.14 (–0.26 to –0.02)		.02^c^		–0.17 (–0.33 to –0.02)			.03		–0.15 (–0.30 to 0.001)			.05			
	t_2_	0.008 (–0.12 to 0.13)		.90		–0.09 (–0.25 to 0.07)			.27		–0.09 (–0.24 to 0.06)			.25			
	Age	–0.0004 (–0.01 to 0.01)		.92		0.01 (0.004 to 0.02)			.005^c^		0.009 (0.001 to 0.02)			.03			
	Gender	–0.22 (–0.42 to –0.02)		.03		0.05 (–0.21 to 0.30)			.72		0.07 (–0.16 to 0.29)			.55			
**Composite stress measure**																	
	t_0_	–0.38 (–0.45 to –0.31)		<.001^c^		–0.38 (–0.46 to –0.30)			<.001^c^		–0.32 (–0.40 to –0.25)			<.001^c^			
	t_1_	–0.11 (–0.19 to –0.04)		.004^c^		–0.11 (–0.20 to –0.03)			.01		–0.07 (–0.15 to 0.01)			.10			
	t_2_	–0.02 (–0.09 to 0.06)		.66		–0.05 (–0.14 to 0.03)			.23		–0.09 (–0.18 to –0.01)			.03			
	Age	–0.001 (–0.01 to 0.01)		.71		0.01 (0.004 to 0.02)			.003^c^		0.01 (0.003 to 0.02)			.002^c^			
	Gender	–0.12 (–0.27 to 0.03)		.12		0.07 (–0.12 to 0.27)			.47		–0.01 (–0.17 to 0.14)			.88			

^a^Time point t_–1_ (ie, baseline) serves as reference category; effect of female gender is depicted.

^b^Missing cases: n_individuals_=30; n_prompts_=1182.

^c^Significant after Simes correction.

^d^Missing cases: n_individuals_=348; n_prompts_=7680.

#### Recovery Period Within Groups Controlled for Subsequent Stressors

When controlling for subsequent stressors in the within-group analysis, that is, the presence or absence of a stressor at the time points after the initial stressor, none of the groups showed a delayed recovery irrespective of the type of stressor. For the composite stress measure, all groups showed a decrease in positive affect at t_0_ compared with t_–1_ (controls: *b*=–0.32, 95% CI –0.40 to –0.25, *P*<.001; at-risk: *b*=–0.38, 95% CI –0.46 to –0.31, *P*<.001; patients: *b*=–0.38, 95% CI –0.45 to –0.31, *P*<.001). At t_1_, all groups had returned to baseline levels of positive affect (controls: *b*=0.02, 95% CI –0.06 to 0.10, *P*=.58; at-risk: *b*=–0.03, 95% CI –0.11 to 0.06, *P*=.53; patients: *b*=0.03, 95% CI –0.04 to 0.11, *P*=.42). Subsequent stress as a control variable was significantly associated with positive affect in all models (controls: *b*=–0.47, 95% CI –0.54 to –0.39, *P*<.001; at-risk: *b*=–0.40, 95% CI –0.48 to –0.32, *P*<.001; patients: *b*=–0.53; 95% CI –0.60 to –0.47, *P*<.001). Similar patterns were found for event-related and activity-related stress (Table 3).

**Table 3:** Within-group analysis of all stress measures comparing positive affect at baseline (t_–1_) with time points t_0_ (stress reactivity), t_1_, and t_2_ (all groups recovered) adjusted for age and gender, and subsequent stress^a^.

Stress type			Patients					At-risk					Controls		
			*b* (CI)		*P* value		*b* (CI)			*P* value		*b* (CI)			*P* value
**Event-related stress^b^**															
	t_0_	–0.36 (–0.43 to –0.29)		<.001^c^		–0.35 (–0.44 to –0.27)			<.001^c^		–0.29 (–0.37 to –0.21)			<.001^c^	
	t_1_	0.07 (–0.01 to 0.15)		.08		–0.02 (–0.11 to 0.08)			.74		0.01 (–0.08 to 0.10)			.82	
	Age	–0.003 (–0.01 to 0.004)		.45		0.01 (0.002 to 0.02)			.01		0.01 (0.004 to 0.02)			.001	
	Gender	–0.11 (–0.26 to 0.04)		.16		0.06 (–0.14 to 0.27)			.55		0.01 (–0.14 to 0.17)			0.87	
	Subsequent stress	–0.54 (–0.61 to –0.47)		<.001^c^		–0.37 (–0.46 to –0.28)			<.001^c^		–0.39 (–0.48 to –0.30)			<.001^c^	
**Activity-related stress^d^**															
	t_0_	–0.49 (–0.60 to –0.38)		<.001^c^		–0.54 (–0.68 to –0.40)			<.001^c^		–0.61 (–0.75 to –0.47)			<.001^c^	
	t_1_	–0.07 (–0.19 to 0.05)		.23		–0.07 (–0.23 to 0.10)			.42		–0.08 (–0.23 to 0.07)			.28	
	t_2_	0.07 (–0.06 to 0.19)		.30		–0.03 (–0.19 to 0.14)			.74		–0.07 (–0.23 to 0.08)			.35	
	Age	–0.001 (–0.01 to 0.01)		.90		0.01 (0.003 to 0.02)			.008		0.009 (0.0002 to 0.02)			.04	
	Gender	–0.21 (–0.41 to –0.01)		.04		0.06 (–0.19 to 0.31)			.65		0.07 (–0.15 to 0.29)			.54	
	Subsequent stress	–0.58 (–0.73 to –0.44)		<.001^c^		–0.66 (–0.87 to –0.45)			<.001^c^		–0.60 (–0.80 to –0.40)			<.001^c^	
**Composite stress measure**															
	t_0_	–0.38 (–0.45 to –0.31)		<.001^c^		–0.38 (–0.46 to –0.31)			<.001^c^		–0.32 (–0.40 to –0.25)			<.001^c^	
	t_1_	0.03 (–0.04 to 0.11)		.42		–0.03 (–0.11 to 0.06)			.53		0.02 (–0.06 to 0.10)			.58	
	t_2_	0.08 (0.01 to 0.16)		.04		0.02 (–0.07 to 0.10)			.72		–0.02 (–0.10 to 0.06)			.65	
	Age	–0.001 (–0.01 to 0.01)		.69		0.01 (0.003 to 0.02)			.004		0.01 (0.003 to 0.02)			.003	
	Gender	–0.11 (–0.26 to 0.04)		.15		0.073 (–0.119 to 0.265)			.45		–0.003 (–0.155 to 0.148)			.966	
	Subsequent stress	–0.53 (–0.60 to –0.47)		<.001^c^		–0.40 (–0.48 to –0.32)			<.001^c^		–0.47 (–0.54 to –0.39)			<.001^c^	

^a^Time point t_–1_ (ie, baseline) serves as reference category; effect of female gender is depicted.

^b^Missing cases: n_individuals_ = 30; n_prompts_ = 1182.

^c^Significant after Simes correction.

^d^Missing cases: n_individuals_ = 348; n_prompts_ = 7680.

### Differences in Baseline (H2), Reactivity (H3), and Recovery (H4) Across Groups

**Figure 1:**
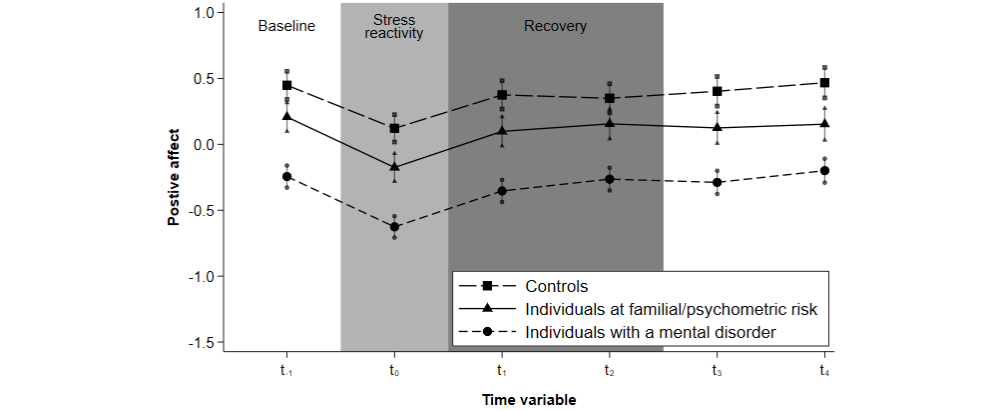
Trajectories of positive affect following composite stress. (Adjusted predictive margins of the multilevel regression analysis for the composite stress measure are displayed. Error bars represent 95% CIs.)

Figure 1 shows the trajectories of positive affect in response to composite stress in all groups. A main effect of group was observed for all stress measures (event-related stress: *χ*^2^_2_=1575.64, *P*<.001; activity-related stress: *χ*^2^_2_=48.69, *P*<.001; composite stress: *χ*^2^_2_=200.9, *P*<.001). There were differences in baseline levels of positive affect across all groups, consistent with H2 (Table 4). However, patients and at-risk individuals did not differ as hypothesized. Patients had significantly lower baseline levels of positive affect than at-risk individuals (*P* values for all stress types <.001). There was no evidence for a 2-way interaction (time_since × group) at t_0_ for any stress measure. This indicated that the associations of event-related stress, activity-related stress, or composite stress with positive affect, that is, the initial positive affective reactivity, did not differ across individuals at different stages of mental disorder, leaving H3 unsupported. As all groups had returned to baseline levels of positive affect by t_2_ following activity-related and composite stress, marking the end point of the continuous recovery period, t_1_–t_2_ were included in the between-group analysis. When examining differences in the average deviation of positive affect from baseline levels during the recovery period t_1_–t_2_ in response to activity-related stress and composite stress, we did not find evidence for between-group differences (Table 4). This indicated that positive affective recovery, operationalized as an average deviation from baseline, was similar across the groups at different stages of mental disorder, leaving H4 unsupported.

**Table 4:** Differences in baseline positive affect (t_–1_), stress reactivity (t_0_), and affective recovery (average deviation of positive affect from baseline levels during t_1_ – t_2_) between groups^a^.

Stress type		At-risk versus controls			Patients versus controls			Patients versus at-risk	
		*b* (95% CI)	*P* value	*b* (95% CI)		*P* value	*b* (95% CI)		*P* value
**Activity stress**									
	t_–1_	–0.34 (–0.57 to –0.11)	.003^b^	–0.74 (–0.95 to –0.54)		<.001^b^	–0.40 (–0.60 to –0.21)		<.001^b^
	t_0_	0.06 (–0.15 to 0.27)	.57	0.11 (–0.07 to 0.29)		.23	0.05 (–0.13 to 0.23)		.58
	t_1_–t_2_	–0.01 (–0.21 to 0.18)	.89	0.05 (–0.12 to 0.22)		.57	0.06 (–0.11 to 0.23)		.46
**Event stress^c^**									
	t_0_	–0.07 (–0.19 to 0.05)	.27	–0.09 (–0.19 to 0.03)		.13	–0.02 (–0.13 to 0.09)		.76
**Composite stress**									
	t_–1_	–0.28 (–0.43 to –0.13)	<.001^b^	–0.44 (–0.57 to –0.31)		<.001^b^	–0.44 (–0.57 to –0.31)		<.001^b^
	t_0_	–0.06 (–0.17 to 0.06)	.35	–0.06 (–0.16 to 0.05)		.30	0.001 (–0.10 to 0.11)		.99
	t_1_–t_2_	0.01 (–0.10 to 0.12)	.89	0.02 (–0.07 to 0.12)		.62	0.02 (–0.08 to 0.11)		.74

^a^Adjusted for age and gender.

^b^Significant after Simes correction.

^c^Model for t_–1_ did not converge.

## Discussion

### Principal Findings

This study aimed to investigate trajectories of positive affect in response to daily life stress across different transdiagnostic clinical stages in a pooled sample of patients with a mental disorder, individuals at psychometric or familial risk, and controls. All groups showed a similar trajectory of positive affect in response to momentary stress, as indicated by a decrease in positive affect to event-related, activity-related, or composite stress, and a continuously lower level of positive affect before recovering to baseline level in response to activity-related or composite stress (H1). We observed a continuous recovery period of 180 minutes on average in patients and at-risk individuals, whereas controls required 90 minutes on average to recover. Comparisons across groups revealed that patients with a mental disorder and at-risk individuals had lower baseline levels of positive affect in daily life compared with controls (H2). Contrary to our prediction, patients had lower levels of positive affect compared with at-risk individuals. Differences in positive affective reactivity to daily stress between groups (H3) and in positive affective recovery fell short of statistical significance (H4).

### Methodological Considerations

Several methodological considerations should be taken into account when interpreting the reported findings. First, because this study used existing data, participants with different clinical characteristics were pooled to form transdiagnostic groups as an approximation to representing subclinical and clinical stages of mental disorder based on the literature of clinical staging. To further support the staging approach and ensure that participants with different clinical characteristics form a group regarding severity of symptoms or functional impairment as suggested by clinical staging, latent class analysis may be used in future analysis to identify groups with similar behavioral patterns. Furthermore, participants may be recruited according to recently developed criteria for clinical staging as there is first evidence for their validity as a way of identifying individuals in early stages with predictive power for transition between stages [3].

Second, the dichotomous operationalization of stress as the presence or absence of a stressor does not account for the degree of unpleasantness of a reported activity or event, which reduces variance. An activity or event rated as –3 may impact positive affect longer than an activity or event rated as –1. Similarly, Vaessen et al [45] showed that emotional reactivity to mild, but not intermediate or strong stressors was related to symptom levels in adolescents 1 year later, suggesting that the degree of unpleasantness of a stressor may need to be accounted for in future studies on affective recovery.

Third, stress reactivity at t_0_ was modeled in a cross-sectional manner, that is, ratings of stress and positive affect measured at the same time point were used to define stress reactivity. Therefore, temporal order between the first stressor of a day and an associated decrease in positive affect remains unclear as a stressor may lead to a decrease in positive affect, or vice versa. Yet, the cross-sectional modeling does not restrict interpretations regarding the recovery period, which was of main interest in this study, operationalized using time points chronologically before and after the occurrence of stress.

Relatedly, the exploratory finding that positive affective recovery within groups may be accounted for by cumulative stress at the following time points should be interpreted with caution. A recent review showed that experiencing positive affect can impact the neural signaling of stress, which may lead to less self-reported stress [46]. As the temporal order between cumulative stress and positive affect measured at the same time point remains unclear, it may, in turn, be possible that being in the recovery period, that is, in a state of decreased positive affect, may lead participants to report more stress.

Last, the composite stress measure combining event- and activity-related stress may hold restrictions. Both stress types may be related to affective recovery in different ways. Specifically, event-related stress is a retrospective judgment of the most important event that happened since the last prompt. As the time points were approximately 90 minutes apart, the unpleasant event might have happened up to 90 minutes before the rating, meaning that an immediate drop in positive affect after the event and the beginning of the recovery period might not have been recorded by the random sampling procedure. Activity-related stress, by contrast, measures the unpleasantness of the current activity. The sampling procedure does not reveal when an unpleasant activity started or for how long it was continued after the measurement, which may also influence positive affect ratings at baseline or during the recovery period. We found no recovery period after event-related stress and effect sizes were lower at t_0_ for event-related stress than at the same time point for activity-related stress (Table 2), indicating that the recovery period for event-related stress may have already begun before reporting the event. Taken together, the sampling procedure in this study may have been limited in detecting differences in positive affective recovery between groups. For future research, a design with more frequent measurements or a hybrid event- and time-contingent sampling procedure may provide more fine-grained modeling of affective recovery.

### Comparison to Prior Research

In line with previous research [15,47], our study showed that levels of positive affect differed between individuals with a mental disorder, individuals at-risk for developing a mental disorder, and controls across the continuum of mental health, thus broadening findings to a transdiagnostic staging approach for the first time. While all groups reported stress reactivity and a period of affective recovery in response to activity-related and overall stress that was descriptively longer within the patient group and at-risk individuals, these differences fell short of statistical significance in between-group analysis comparing average deviations of positive affect from baseline levels. Yet, levels of positive affect in patients and at-risk individuals were generally lower across the entire recovery period (Figure 1). This may suggest that reactivity of similar magnitude and a recovery period of similar length may be associated more strongly with risk and disorder when operating on lower overall levels of positive affect.

Furthermore, the magnitude of differences in positive affective reactivity and recovery between groups might have been too small to be detected with the number of observations per day in our models. In addition, criteria other than clinical status might be relevant to index risk and identify group differences in trajectories of positive affect in response to minor stressors, such as childhood adversities. In line with the stress sensitization hypothesis [12], stress reactivity as a behavioral marker for stress sensitization has been shown to be amplified in individuals exposed to severe adversity across the life course [22,48-51]. For instance, stress reactivity in early and later stages of psychopathology has been reported to be greater in individuals exposed to high levels of childhood adversity than in controls exposed to high levels of adversity, suggesting they were more resilient [22]. Future research should investigate whether this holds true for differences in positive affective recovery as a transdiagnostic marker for momentary resilience, that is, the ability to recover from minor stressors in the moment. Differences in affective recovery across stages may only become evident when viewed in the context of exposure to adversities across the life course.

To our knowledge, this is the first study to transdiagnostically investigate the trajectories of positive affect after minor stressors in daily life. It has been shown that positive and negative affect can be conceptualized as 2 distinct factors [52] that are related to positive and negative events in daily life in different ways. For example, negative events were found to be less strongly related to positive than to negative affect [53]. Similarly, Wichers et al [54] found that physical activity, which may be regarded as a positive activity, was related to momentary positive affect, but unrelated to momentary negative affect. Adding to previous findings [20], we found a shorter period of positive affective recovery after a negative event than was found for negative affective recovery after a negative event. Furthermore, group differences in negative affective recovery between at-risk individuals and individuals with a mental disorder were not reflected in our findings regarding positive affective recovery. Taken together, this may suggest that the trajectories of positive and negative affect in response to minor daily stressors constitute separate psychological mechanisms. This underlines the differential role of positive affect for psychological well-being [24,27] and highlights the need to investigate, in more detail, how positive and negative affective recovery compare in stages of mental disorder.

### Implications

In this study, we found first evidence for different trajectories of positive affect following minor daily stressors in a transdiagnostic sample covering the continuum of mental health. Whether positive affective recovery on the scale of minor stressors in daily life may be a putative indicator for momentary resilience should be investigated further in the context of childhood adversity, specifically focusing on healthy, that is, resilient, individuals exposed to adversities. When disentangling this putative protective mechanism further, trajectories of affective recovery may potentially serve as a target for ecological momentary interventions, a mobile health approach using mobile devices to deliver interventions in daily life [55,56]. Targeting affective recovery, intervention components may potentially be presented in moments when participants experience stress helping them to recover, and ultimately foster resilience in early and later stages of psychopathology. Targeting this putative momentary mechanism in ecological momentary interventions allowing the use of experimental designs in daily life [57] may allow us to understand more fully the role of affective recovery in pathways to severe mental disorder. This will provide evidence for the effectiveness and feasibility of scalable interventions for transdiagnostic populations.

## Appendix

Multimedia Appendix 1 Graphic illustration of the expected trajectories of positive affect in the context of a minor daily stressor in subclinical and clinical stages of mental disorder.

Multimedia Appendix 2 Overview of pooled studies.

Multimedia Appendix 3 Principle component analysis of the composite stress measure.

Multimedia Appendix 4 Unadjusted within-group analysis comparing positive affect at baseline (t_-1_) to time points t_0_ (stress reactivity) to t_n_ (all groups recovered).

## Abbreviations

ESM: experience sampling methodology

## Data Availability

Data pertain to the ESM merge file. The ESM merge file is a data set combining multiple ESM studies that have been performed at Maastricht University. Data are available upon reasonable request from researchers. Researchers can send their request to the Department of Psychiatry & Neuropsychology of Maastricht University, School for Mental Health and Neuroscience, by sending an email to info@esm-maastricht.nl.
