# Supplementary material for: Positive Affective Recovery in Daily Life as a Momentary Mechanism Across Subclinical and Clinical Stages of Mental Disorder: Experience Sampling Study
Source: JMIR Ment Health. 2022 Nov 23;9(11):e37394. doi: 10.2196/37394 (PMC9730210; doi:10.2196/37394)
Supplement: Multimedia Appendix 1 [file mental_v9i11e37394_app1.docx]

Figure 1. Graphic illustration of the expected trajectories of positive affect in the context of a minor daily stressor in subclinical and clinical stages of mental disorder.


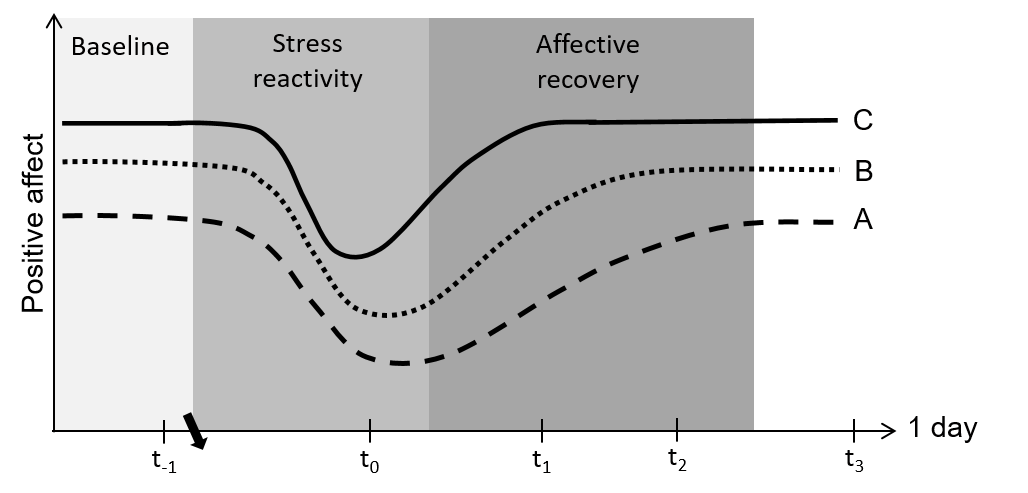


*Note.* A = individuals with a mental disorder; B = individuals at familial or psychometric risk for a mental disorder; C = controls; minor stressor in daily life represented by black arrow on the x-axis; time points of experience sampling methodology (ESM) ranging from t-1 = time point before stressor was reported, t0 = time point, at which stressor was reported, t1 to t2 = time points making up the recovery period, to t3 = third time point after stressor was reported, at which all groups have recovered to baseline level positive affect.
