# Supplementary material for: Positive Affective Recovery in Daily Life as a Momentary Mechanism Across Subclinical and Clinical Stages of Mental Disorder: Experience Sampling Study
Source: JMIR Ment Health. 2022 Nov 23;9(11):e37394. doi: 10.2196/37394 (PMC9730210; doi:10.2196/37394)
Supplement: Multimedia Appendix 2 [file mental_v9i11e37394_app2.docx]

Table 1. Overview of pooled studies.

| Study | N | Criteria/ Description |
| --- | --- | --- |
| **Apriprazole  [33]** | N = 27  Patients | Patients   1. Diagnosis of schizophrenia displaying insufficient therapeutic response to antipsychotic treatment |
| **PREVENT  [19]** | N = 54  At-risk (n = 27); Controls (n = 27) | High-risk   1. Attenuated positive symptoms assessed with the Structured Interview for Prodromal Symptoms (SIPS) 2. brief limited intermittent psychotic symptoms (BLIPS) assessed with SIPS 3. Presence of at least two basic symptoms (e.g., subtle thought and speech disturbances) assessed with Schizophrenia Prediction Instrument, Adult version (SPI-A) 4. A significant drop in functioning (30% on the Global Assessment of Functioning (GAF) for at least 1 month within the last year) in combination with genetic risk; or 5. A significant drop in functioning plus a diagnosis of schizotypal personality disorder   Controls   1. Healthy controls |
| **MACS  [35]** | N = 18  Patients | Patients   1. DSM-IV Diagnosis of schizophrenia |
| **MAPS  [17]** | N = 146  Patients (n = 48); At-risk (n = 48); Controls (n = 50) | Patients   1. Lifetime occurrence of psychotic symptoms (according to Research Diagnostic Criteria) for at least two weeks in clear consciousness   High-risk   1. Relative of a psychotic patient with no history of psychotic symptoms   Controls   1. Neither family nor personal history of psychosis |
| **MindMaastricht  [39]** | N = 129  Patients | 1. Residual symptomatology defined as a score of seven or higher on the Hamilton Depression Rating Scale (HDRS) after at least one episode of Major Depressive Disorder |
| **GROUP  [36, 37]** | N = 238  Patients (n = 73); At-risk (n = 81); Controls (n = 84) | Patients   1. Diagnosis of a non-affective psychotic disorder   High-risk   1. Sibling of patient with non-affective psychotic disorder   Controls   1. Community sample |
| **STRIP  [34, 40]** | N = 148  Patients (n = 48); At-risk (n = 49); Controls (n = 51) | Patients   1. Diagnosis of a psychotic disorder   High-risk   1. Healthy first-degree relatives of individuals with a psychotic disorder   Controls   1. No history of psychiatric illness according to the explicit diagnostic criteria of the DSM-IV-TR 2. no family history of psychotic illness |
| **ZAPP  [38]** | N = 158  Patients (n = 79); At-risk (n = 41); Controls (n = 38) | Patients   1. Diagnosed with a psychotic with paranoid psychotic symptoms, defined as having a score of > 3 on Item P6 (suspiciousness) of the Positive and Negative Syndrome Scale (PANSS) 2. Diagnosed with a psychotic disorder who currently have other positive psychotic symptoms, defined as having a score of < 4 on the PANSS Items P6 (suspiciousness) and having a score of > 3 on at least one of the PANSS Items P1 (delusions), P3 (hallucinatory behavior), P5 (grandiosity), and G9 (unusual thought content) 3. Diagnosed with a psychotic disorder currently reporting remitted psychotic symptoms, defined as having a score of < 4 on all the aforementioned PANSS items   High-risk   1. No diagnosis of psychotic disorder presenting a psychometric at-risk mental state for paranoid psychosis defined operationally as scoring high (> 90th percentile) on the paranoid items of a questionnaire measuring psychosis-proneness (Community Assessment of Psychic Experiences, CAPE)   Controls   1. Low schizotypy: scoring in the average range (between the 45th and 55th percentile) on all three symptom dimensions of the CAPE and not scoring high (> 90th percentile) on the paranoid items |
