# Supplementary material for: Positive Affective Recovery in Daily Life as a Momentary Mechanism Across Subclinical and Clinical Stages of Mental Disorder: Experience Sampling Study
Source: JMIR Ment Health. 2022 Nov 23;9(11):e37394. doi: 10.2196/37394 (PMC9730210; doi:10.2196/37394)
Supplement: Multimedia Appendix 3 [file mental_v9i11e37394_app3.docx]

Appendix 3. Principle component analysis of the composite stress measure.

Principle component analysis was performed to justify the use of a composite stress measure. The Kaiser-Meyer-Olkin measure of sampling adequacy was .50, which is the acceptable limit by Kaiser [1]. Bartlett’s test of sphericity was significant (χ^2^(1)=229.14, p<.001), indicating that the stress measures were sufficiently intercorrelated. One factor exceeding an eigenvalue of 1 was retained explaining 56.10% of the variance. The composite stress measure was, therefore, used in the analysis.

**Reference**

1. Kaiser HF. An index of factorial simplicity. Psychometrika. 1974;39 (1):31-6.
