# Supplementary material for: Positive Affective Recovery in Daily Life as a Momentary Mechanism Across Subclinical and Clinical Stages of Mental Disorder: Experience Sampling Study
Source: JMIR Ment Health. 2022 Nov 23;9(11):e37394. doi: 10.2196/37394 (PMC9730210; doi:10.2196/37394)
Supplement: Multimedia Appendix 4 [file mental_v9i11e37394_app4.docx]

Table 1. Unadjusted within-group analysis comparing positive affect at baseline (t_-1_) to time points t_0_ (stress reactivity) to t_n_ (all groups recovered).

|  | Patients | | At-risk | | Controls | |
| --- | --- | --- | --- | --- | --- | --- |
|  | *b* [CI] | *P* | *b* [CI] | *P* | *b* [CI] | *P* |
|  |  |  |  |  |  |  |
| **Composite stress measure** |  |  |  |  |  |  |
| t_0_ | -.38  [-.45; -.31] | <.001 | -.38  [-.46; -.30] | <.001 | -.32  [-.40; -.25] | <.001 |
| t_1_ | -.11  [-.19; -.04] | 0.004 | -.11  [-.20; -.03] | 0.009 | .07  [-.15; .01] | 0.097 |
| t_2_ | -.017  [-.09; .06] | 0.66 | -.05  [-.14; .04] | 0.25 | -.09  [-.18; -.01] | 0.03 |
| **Event-stress only^a^** |  |  |  |  |  |  |
| t_0_ | -.35  [-.43; -.28] | <.001 | -.34  [-.43; -.26] | <.001 | -.27  [-.35; -.19] | <.001 |
| t_1_ | -.06  [-.13; .02] | 0.16 | -.07  [-.16; .02] | 0.10 | -.04  [-.13; .04] | 0.32 |
| **Activity-stress only^b^** |  |  |  |  |  |  |
| t_0_ | -.49  [-.61; -.38] | <.001 | -.54  [-.68; -.40] | <.001 | -.60  [-.74; -.46] | <.001 |
| t_1_ | -.14  [-.26; -.02] | 0.02 | -.17  [-.33; -.02] | 0.03 | -.15  [-.30; .0007] | 0.05 |
| t_2_ | .01  [-.12; .13] | 0.91 | -.09  [-.25; .07] | 0.28 | -.09  [-.24; .06] | 0.25 |

*Note.* Time point t_-1_ (i.e., baseline) serves as reference category.

^a^ Missing cases: N_individuals_ = 30; N_prompts_ = 118

^b^ Missing cases: N_individuals_ = 348; N_prompts_ = 7680
